# Comparative Innate and Adaptive Immune Responses in Atlantic Bottlenose Dolphins (*Tursiops truncatus*) With Viral, Bacterial, and Fungal Infections

**DOI:** 10.3389/fimmu.2019.01125

**Published:** 2019-05-29

**Authors:** Gregory D. Bossart, Tracy A. Romano, Margie M. Peden-Adams, Adam M. Schaefer, Charles D. Rice, Patricia A. Fair, John S. Reif

**Affiliations:** ^1^Georgia Aquarium, Atlanta, GA, United States; ^2^Division of Comparative Pathology, Miller School of Medicine, University of Miami, Miami, FL, United States; ^3^The Mystic Aquarium, a Division of Sea Research Foundation, Inc., Mystic, CT, United States; ^4^Harry Reid Center for Environmental Studies, University of Nevada, Las Vegas, NV, United States; ^5^Harbor Branch Oceanographic Institute at Florida Atlantic University, Ft. Pierce, FL, United States; ^6^Graduate Program in Environmental Toxicology, Department of Biological Sciences, Clemson University, Clemson, SC, United States; ^7^Department of Public Health Sciences, Medical University of South Carolina, Charleston, SC, United States; ^8^Department of Environmental and Radiological Health Sciences, College of Veterinary Medicine and Biomedical Sciences, Colorado State University, Fort Collins, CO, United States

**Keywords:** bottlenose dolphin, *Tursiops truncatus*, innate immune response, adaptive immune response, infectious disease

## Abstract

Free-ranging Atlantic bottlenose dolphins (*n* = 360) from two southeastern U.S. estuarine sites were given comprehensive health examinations between 2003 and 2015 as part of a multi-disciplinary research project focused on individual and population health. The study sites (and sample sizes) included the Indian River Lagoon (IRL), Florida, USA (*n* = 246) and Charleston harbor and associated rivers (CHS), South Carolina, USA (*n* = 114). Results of a suite of clinicoimmunopathologic tests revealed that both populations have a high prevalence of infectious and neoplastic disease and a variety of abnormalities of their innate and adaptive immune systems. Subclinical infections with cetacean morbillivirus and *Chlamydiaceae* were detected serologically. Clinical evidence of orogenital papillomatosis was supported by the detection of a new strain of dolphin papillomavirus and herpesvirus by molecular pathology. Dolphins with cutaneous lobomycosis/lacaziasis were subsequently shown to be infected with a novel, uncultivated strain of *Paracoccidioides brasiliensis*, now established as the etiologic agent of this enigmatic disease in dolphins. In this review, innate and adaptive immunologic responses are compared between healthy dolphins and those with clinical and/or immunopathologic evidence of infection with these specific viral, bacterial, and fungal pathogens. A wide range of immunologic host responses was associated with each pathogen, reflecting the dynamic and complex interplay between the innate, humoral, and cell-mediated immune systems in the dolphin. Collectively, these studies document the comparative innate and adaptive immune responses to various types of infectious diseases in free-ranging Atlantic bottlenose dolphins. Evaluation of the type, pattern, and degree of immunologic response to these pathogens provides novel insight on disease immunopathogenesis in this species and as a comparative model. Importantly, the data suggest that in some cases infection may be associated with subclinical immunopathologic perturbations that could impact overall individual and population health.

## Introduction

Emerging infectious disease has become a complex and serious concern that has consequences for human, animal, and environmental health on a global scale (1, 2). As in terrestrial species, emerging infectious agents in marine mammals, particularly Atlantic bottlenose dolphins (*Tursiops truncatus*), may be associated with neoplasia, epizootics, and zoonotic transmission to humans. These emerging diseases are characterized by a multifactorial etiology involving an infectious agent and non-infectious cofactors including organic and inorganic contaminants, biotoxins, and other environmental stressors (3–5). The immune system plays a pivotal role in the pathogenesis and outcome of infectious disease. However, unanswered questions remain regarding the structure and function of the immune system in dolphins. Specifically, the immunopathogenesis of newly characterized infectious diseases and related protective immunity have not been characterized fully. A positive outcome (absent or reduced morbidity, survival) following infection depends on the host's ability to mount a diversified immunologic response to the pathogen. Understanding the mechanisms, interactions, and events that affect the innate and adaptive immune systems is a critical first step in understanding the dynamics of infectious disease in free-ranging marine mammals. Answering these questions is especially important for the dolphins that inhabit the Indian River Lagoon, Florida, USA (IRL) and the Charleston, South Carolina, Harbor and related estuarine riverine ecosystem USA (CHS). A high prevalence of disease has been detected for both populations, the majority of which is caused by infectious agents (3, 6). To further characterize the dolphins' immune response to infectious and non-infectious environmental stressors we incorporated a suite of measurements to evaluate the innate and adaptive immune systems during health assessments of these two populations. The purpose of this review is to compare the innate and adaptive immunologic responses in healthy dolphins with dolphins that have clinicopathologic or serologic evidence of infection by specific viral, bacterial, and fungal pathogens.

## Materials and Methods

### Background

The multidisciplinary, multi-institutional Atlantic Bottlenose Dolphin Health and Environmental Risk Assessment (HERA) Project was initiated in 2003 to evaluate individual and population health in two southeastern USA estuarine locales: the IRL and coastal waters of CHS (3). A major project goal was to further develop classical and newer diagnostic methods for the evaluation of dolphin health. Further, we aimed to conduct environmental risk assessment by exploring the relationships between health status and a variety of environmental stressors of anthropogenic and natural origin. Bottlenose dolphins serve as a sentinel species for environmental and public health due to their residence in coastal ecosystems, longevity, and high trophic level status (2). As a component of their role as sentinels, studies of bottlenose dolphins may be pivotal in assessing the role of chemical agents and toxins in infectious diseases, particularly through modulation of the immune system.

### Study Sites

**Figure 1:**
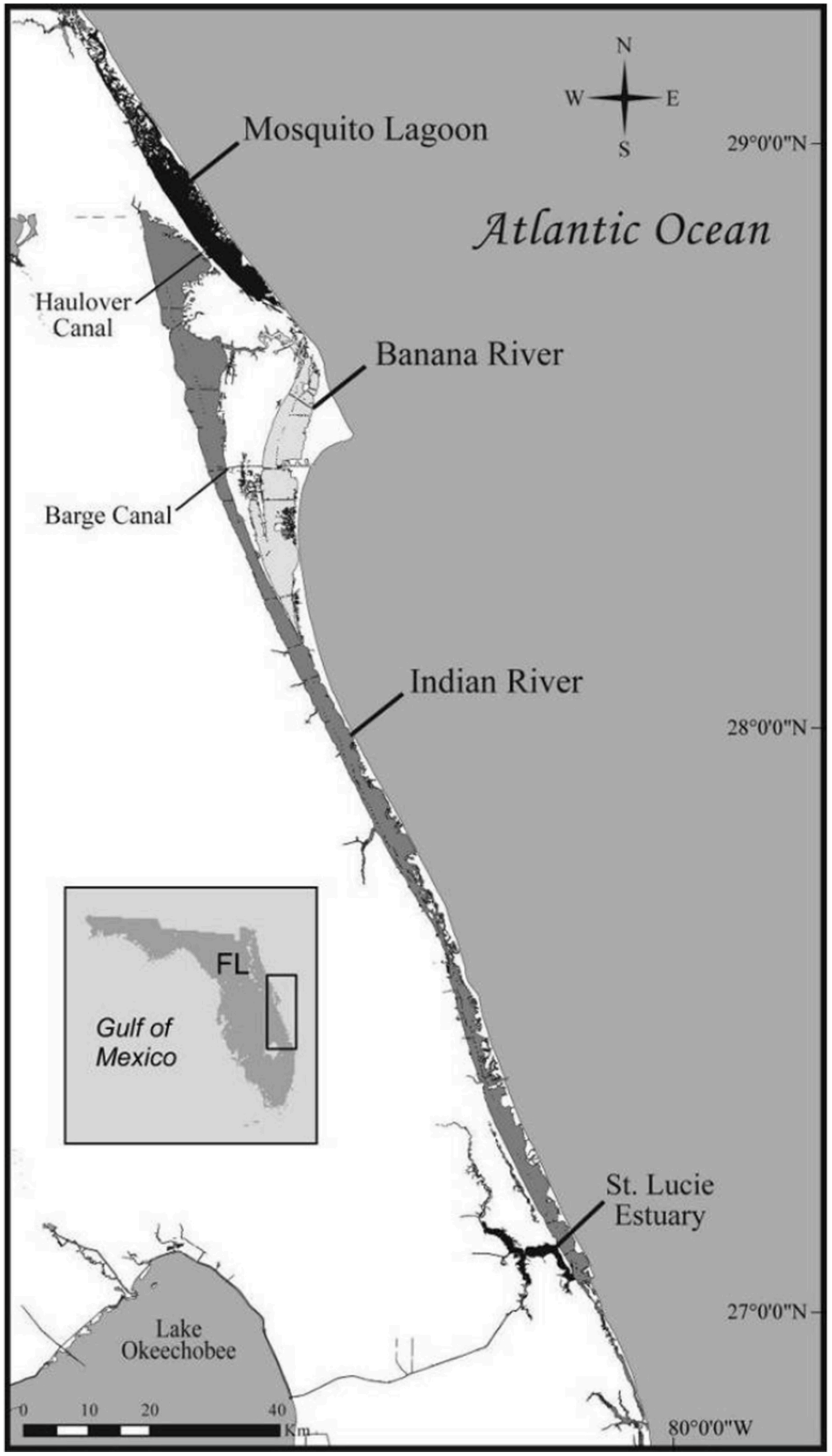
The Indian River Lagoon, Florida (USA) study site.

Spatial descriptions of the IRL and CHS study sites are provided in detail in previous reports (4, 7). The IRL is a 250 km longitudinal shallow-water estuary formed by the aggregate of the Indian River, the Banana River, and the Mosquito Lagoon (Figure 1). The IRL was designated an Estuary of National Significance since it is the most biodiverse estuary in North America (8). Residential development, agricultural activities, and runoff and fresh water inputs from drainage canals have adversely impacted water quality in the IRL in recent years.

Early studies of health in IRL dolphins were based on pathologic findings from deceased dolphins. These reports suggested that a substantial component of mortality was due to infectious diseases and that immunologic dysfunction may have played a role in pathogenesis (9). Unusual Mortality Events (UMEs) in IRL bottlenose dolphins were declared in 2001, 2008, and 2013 in which the number of observed deaths significantly exceeded expected rates and for which no specific cause could be established (10, 11). More recently, a UME due to an epizootic of cetacean morbillivirus (CeMV; see below) caused the deaths of approximately 1,650 dolphins along the Mid-Atlantic coast including the IRL (11, 12).

**Figure 2:**
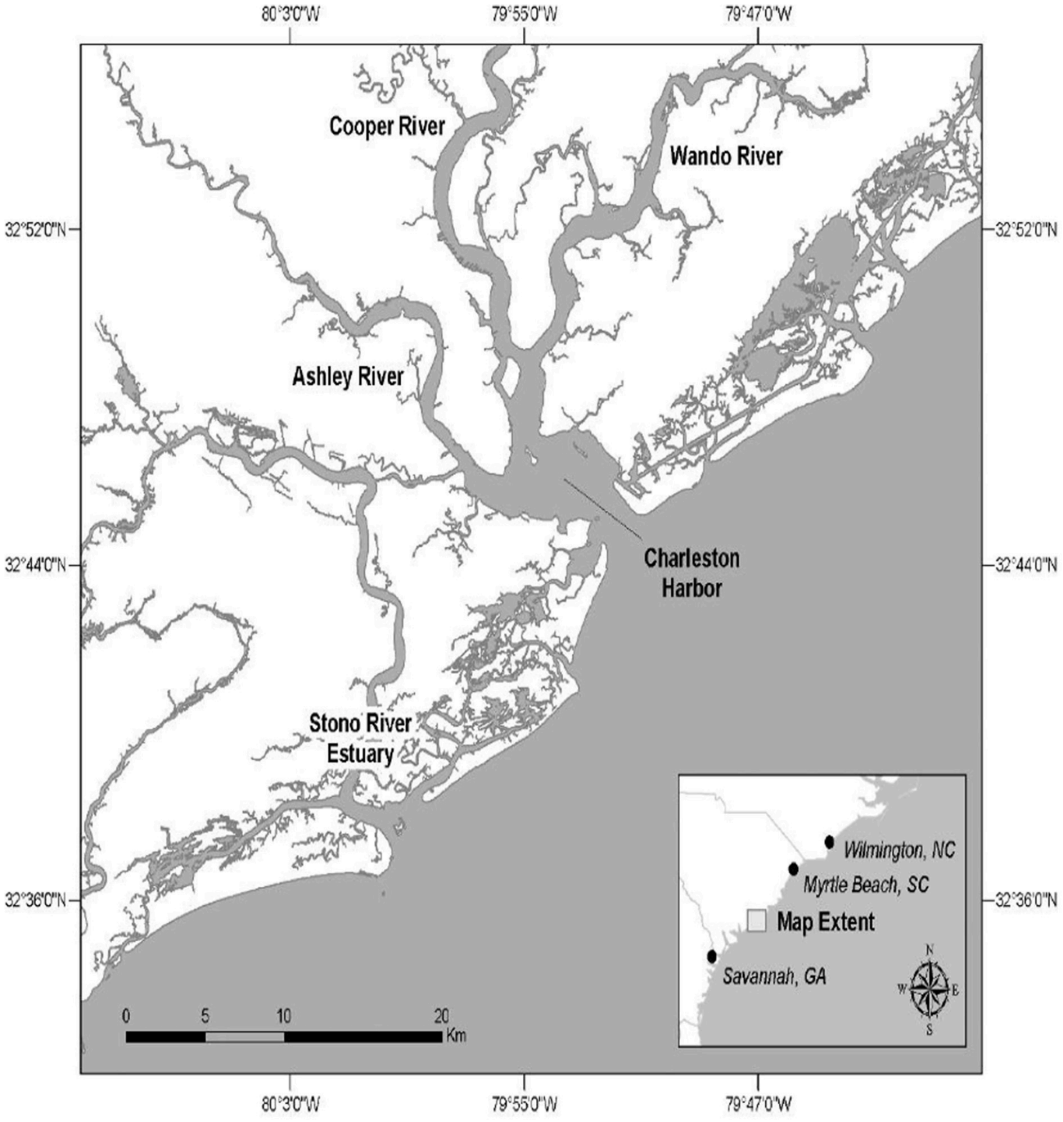
The Charleston, South Carolina (USA) study site.

The CHS study site is comprised of portions of the Charleston Harbor estuary and related rivers and the Stono River estuary (Figure 2).

Bottlenose dolphins comprised 73% of marine mammal strandings in South Carolina between 1992 and 1996. Carcasses were recovered primarily in the CHS study area (13). In an analysis of mortality, 31% of dolphin deaths in the CHS area were shown to be due to infectious diseases, while a large component (47%) was attributed to non-infectious causes such as human interaction by trauma, net entanglement etc. (14). Both the IRL and CHS dolphin populations display site fidelity characterized by long-term residency patterns. Photo identification data have used to estimate that the IRL contains a population of ~1,000 dolphins (15) and the CHS population ranges seasonally and annually between ~200 and 650 (16). Site fidelity is an important population attribute that allows analysis of spatial and temporal trends in disease prevalence.

### Dolphin Health Assessments

The HERA project was conducted in the IRL during June 2003 to 2007, 2010 to 2012, and in 2015. Dolphins were sampled at the CHS site during August of 2003 to 2005, and in 2013. A well-defined protocol was used in all samplings to assure that the conditions of capture, examination, sample collection, processing, and storage were conducted under standardized conditions at each site and during each year (7). Full details were published previously (7).

Health examinations consisted measurement of body weight and morphometric parameters, monitoring of vital signs, a complete physical examination, and ultrasound examination of females to determine pregnancy status. Biologic samples were collected for the measurement of the following parameters: blood (complete blood count, serum chemistry, serum protein electrophoresis, serology, immunologic parameters; see below for details); gastric fluid, feces, and nasal sinus exudate (cytologic evaluation, microbiology, antibiotic sensitivity testing); urine (standard urinalysis) blubber, skin, and lesion biopsies (virology, histopathology, organic and inorganic contaminants). See previous publications for further detail (6, 17–26).

Dolphin health status was categorized using clinicopathologic data into three groups (25). Clinically healthy dolphins had no abnormalities on physical examination or by screening hematology and serum chemistry results. Possibly diseased dolphins had abnormalities which would require further testing and observation under veterinary care. Diseased dolphins had one or more abnormalities which would require medical treatment if the dolphin was housed in a managed-care setting under veterinary supervision. The standard method to determine dolphin age was used which consists of counting dentine layers in an extracted tooth (27). Alternatively, age was estimated by using reference ranges for total body length as published previously when a tooth could not be obtained (28). Dolphin age was classified as follows: adults > 6 yr. sub adults 3.5–5.9 yr (17). Calves were not captured; females determined to be pregnant by ultrasound examination were released immediately as specified by permit regulations. Research was conducted under US National Marine Fisheries Service Scientific Research Permit Nos. 998-1678 and 14352 issued to G. Bossart and Florida Atlantic University IACUC protocol number A10-13.

### Blood Collection

Whole blood and serum samples were collected from the periarterial venous fluke rete and prepared as previously described (17–19, 29). Samples for total and differential white blood cell counts (WBC) and serum protein electrophoresis were processed by the Cornell University Veterinary Diagnostic Laboratory in Ithaca, New York, USA. Blood samples for specific immunologic assays were processed by the Mystic Aquarium, a division of Sea Research Foundation, Mystic, Connecticut, USA and the NOAA Ocean Service, Charleston, South Carolina, USA.

### White Blood Cell Hematology and Serum Protein Electrophoresis

The total WBC count and WBC differential count provide classical clinicopathologic information including the total number and types of peripheral blood granulocytic and mononuclear leukocytes that are important in both the innate and adaptive immune responses (30). Serum protein electrophoresis is used to identify changes in alpha, beta, and gamma globulins that are produced in various inflammatory and infectious disease states in dolphins (31). Specifically, elevated alpha/beta globulins and elevated gamma globulins typically reflect an acute phase protein response and antigenic stimulation of humoral immunity, respectively (3, 31).

Total WBCs were determined by an automated analyzer (Bayer ADVIA 120, Bayer Diagnostics) and the relative number of WBC types were determined by microscopic examination of modified Wright-Giemsa stained blood smears (18). Serum protein electrophoresis was evaluated on an automated analyzer (Rapid Electrophoresis, Helena Laboratories) as previously described (31).

### Immune Assays

The methods used for the determination of innate and adaptive immune system tests including the sources of reagents and antibodies were previously characterized in detail (18, 25), Briefly, peripheral blood leukocytes (PBLs) were isolated, enumerated, and evaluated for viability for assessment of NK-cell activity, immunophenotyping, and lymphocyte proliferation (32).

### Phagocytosis, Lysozyme, and Natural Killer Cell (NK) Activity

Phagocytosis, lysozyme and NK activity were specific innate immune system indicators evaluated in this study. Granulocytic and monocyte phagocytosis is the principal mechanism for the initial destruction of invading or foreign antigens and as such makes up an important innate immune system component (33). A suppression of granulocytic or monocyte phagocytosis can result in increased susceptibility to bacterial and fungal infections. The percent phagocytosis for granulocytes and monocytes was determined using a technique previously described (34). One hundred thousand gated granulocytes and monocytes were analyzed for percent phagocytosis on an LSR flow cytometer (BD Biosciences, San Jose, CA, USA) by histogram statistics (34).

Lysozyme is an enzyme that catalyzes the hydrolysis of some bacterial cell walls causing lysis of the bacteria, (35). Lysozyme activity was assessed using slight modifications of a standard turbidity assay described previously (32, 36).

Natural killer cell activity is the innate immune system's principal mechanism for killing neoplastic cells and virus-infected cells in the early stages of infection (37). NK cell activity was assessed via an *in vitro* cytotoxicity assay as described previously with slight modifications (38).

### Immunophenotyping and Mitogen-Induced Lymphocyte Proliferation (LP)

Immunophenotyping and mitogen-induced LP were performed to help characterize the structure and function of the dolphin's adaptive immune response. Immunophenotyping was used to differentiate specific types of immune cells and the proteins expressed by these cells in the adaptive immune response. Lymphocyte subsets were labeled and analyzed according to methods described previously (6, 18, 39–44). Lymphocytes were analyzed by a LSR flow cytometer (BD Biosciences, San Jose, CA, USA). Ten thousand lymphocyte-gated events were evaluated by histogram statistics (44).

LP is the first step in a functional adaptive immune response to create effector lymphocytes necessary for T cell and B cell mediated immune responses (37). The LP response was measured using techniques optimized previously (45). Briefly, isolated viable PBLs were incubated in well plates with concanavalin A (Con A; a T-cell mitogen), lipopolysaccharide (LPS; E. coli 055:B5; a B-cell mitogen), or supplemented RPMI-1640 representing unstimulated wells in triplicate followed by the addition of tritiated thymidine. Cells were then harvested and assessed using a scintillation counter (Packard, Meriden, CT, USA).

### Antibody Titers Against Marine Bacteria

Antibody titers against common marine bacteria were determined by a previously validated ELISA technique which was used to assess a general humoral response to common marine pathogens (17, 46). Cultures of *Escherichia coli, Erysipelothrix rhusiopathiae, Mycobacterium marinum, Vibrio cholerae, Vibrio carchariae, Vibrio vulnificus*, and *Vibrio parahemolyticus* were acquired from the American Type Culture Collection (ATCC, Manassas, VA, USA). Serum ELISA antibody titers from individual dolphins were then expressed as antibody titers at a 1:200 serum dilution (47).

### Cetacean Morbillivirus Serology

The *Morbillivirus* genus of the Paramyxoviridae family includes the marine mammal pathogens of canine distemper virus phocine distemper virus and cetacean morbillivirus (CeMV). Other mammalian viruses in the *Morbillivirus* genus include measles virus in humans and primates, pestes des petits ruminants virus in small ruminants, and rinderpest virus in large ungulates (48).

A serum neutralization test for CeMV was validated and performed at the Veterinary Diagnostic Laboratory, University of Georgia, Athens, Georgia, USA. CeMV was grown in Vero cells and the test was performed as previously reported (18, 49, 50). Briefly, antibody titers were expressed as the reciprocal of the highest serum dilution that completely neutralized virus cytopathic effect. Titers ≥8 were considered positive for morbillivirus neutralizing serum antibody (50).

### Chlamydiaceae Serology

*Chlamydiaceae* comprise a large family of obligate, Gram-negative bacteria that can be the etiology of complex, multisystemic, and zoonotic disease in a wide range of domestic and wildlife species (51, 52). Interestingly, the host immune response may be ineffective in infection resolution-and actually may contribute to progression of the disease (53). Ultimately, the resolution of chlamydial infection is an immunologic challenge considering the bacteria's unique extracellular and intracellular vegetative infectious phases (54). Clinical disease due to *Chlamydiaceae* has not been reported in marine mammals.

An indirect fluorescent antibody (IFA) test was developed and utilized for determining antibody titers to *Chlamydiaceae* at the Avian and Wildlife Laboratory, School of Medicine, University Of Miami, Miami, Florida, USA as previously reported (19, 55). Briefly, *C. trachomatis* was used for its growth characteristics and antigenic similarities which are shared with other species of Chlamydia and Chlamydophila (53, 56). The IFA method was validated with samples from confirmed cases of *C. psittaci* and reported to correlate well with the Chlamydophila elementary body agglutination serology assay and other, alternative serological methods (55, 57). *Chlamydiaceae* titers of >1:50 were considered seropositive. Based on past studies this titer is considered indicative of recent infection, re-infection or chronic infection (19, 58).

### Paracoccidioidomycosis Ceti Diagnostics

Based on historic histopathologic evidence, lobomycosis (or lacaziosis) was identified as a chronic mycotic dermatopathy found naturally only in infected dolphins and humans (59, 60). The presumed zoonotic and causal organism, *Lacazia loboi*, was described as an uncultivated, yeast-like organism found within cutaneous lesions (61). However, recent molecular analysis demonstrates that a novel uncultivated strain of *Paracoccidioides brasiliensis* is the causal organism of dolphin lobomycosis. The disease has been renamed paracoccidioidomycosis ceti (PC) (62).

The diagnosis of PC was based on histopathologic evidence from incisional biopsies of cutaneous lesions that were aseptically collected following local anesthesia (9). Biopsies for histologic evaluation were placed in 10% neutral buffered formalin, processed routinely, cut at 5 μm, and stained with hematoxylin and eosin (H&E) and Gomori methenamine silver (GMS). The gross and microscopic pathologic findings of PC were previously identified (17, 20, 25, 59, 63). Grossly PC is characterized by focal to multifocal, white, raised, and firm cutaneous nodules which may coalesce and progress slowly (59). Microscopically, PC lesions consist of multifocal to coalescing dermal foci of granulomatous inflammation characterized by infiltrates of macrophages, epithelioid cells, multinucleated giant cells, and often large numbers of GMS positive yeast-like cells, 6 to 12 um in diameter, arranged singly or in chains and joined by tubular connections (20, 59, 64).

### Orogenital Papilloma (OP) Diagnostics

In the past 12 years, nine novel bottlenose dolphin papillomaviruses (PV) (TtPV1-9) have been characterized in genital papillomas. These viruses were determined to belong to the genera *Omikronpapillomavirus, Upsilonpapillomavirus*, and *Dyopipapillomavirus* by phylogenetic analysis (65–69). Two delphinid gammaherpesviruses (DeHV-4 and−5) have also been found with PV co-infections (70, 71). Orogenital papillomas associated with novel PV and herpesvirus infections were first described in wild bottlenose dolphins from southeast Atlantic coastal waters in 2004 (3, 20, 63).

Lingual and genital mucosal neoplastic lesions were grossly and microscopically identical benign sessile papillomas. Diagnosis was based on characteristic pathologic findings (20). Incisional biopsies of tongue and genital lesions were aseptically obtained following local anesthesia as described above (63). Biopsies were put in 10% neutral buffered formalin, processed routinely, cut at 5 μm, and stained with H&E. The gross and microscopic pathologic findings of OP were previously identified (17, 20, 63). In short, OP was characterized by focal to multifocal, irregular to circular, raised, soft, white to light pink, non-pedunculated lesions, ranging from 0.5 to 2 cm in diameter. The surfaces of the OP lesions were non-ulcerative and typically soft, smooth or fissured. Microscopically, OP was characterized by focal plaques composed of uniformly hyperplastic and occasionally dysplastic keratinocytes with mild to moderate koilocytosis.

### Study Designs and Statistical Analyses

All statistical analyses and results summarized below are based on the data contained in the original publications for each infectious disease (17–19, 25). A case-control approach was used for each study by selecting apparently healthy dolphins from the HERA database and comparing them to animals affected with each of the conditions. In all analyses, the values for clinical and immune parameters in healthy dolphins were compared with those for affected dolphins from the same population. In instances where an individual dolphin was re-captured in subsequent years, the data from the first capture were used for analysis. The data for CeMV and PC were obtained from the IRL population only since infected animals were not identified at CHS. Analyses for OP and *Chlamydiaceae* seropositivity were conducted for the pooled IRL and CHS populations. Initial evaluation included the calculation of descriptive statistics, range, mean, distribution, and standard deviation (SD) for each clinical and immune parameter. All data were evaluated for normality using the Shapiro-Wilks statistic or the Kolmogorov-Smirnov test for normality. Logarithmic transformation was attempted to provide normal distributions where applicable. The Mann–Whitney *U*-test was applied to serologic data for CeMV and *Chlamydiaceae* which remained non-normally distributed after attempted transformations. The Wilcoxon rank-sum test was used to compare PC and OP histologically positive and negative animals. Potential confounding by age, gender, and site was evaluated in each data set. CeMV and *Chlamydiaceae* data were analyzed with a multivariable ANOVA with adjustment for age. Analyses were conducted in SPSS version 20 (IBM, Armonk, NY) except for PC which was conducted in SAS version 9.1 (SAS Institute, Cary, NC). Statistical significance was defined as *p* < 0.05 in all analyses.

## Results

### Health Status Classification and Disease Prevalence

The HERA study population consisted of 360 dolphins (246 IRL, 114 CHS). The larger sample size for the IRL is attributable to a larger number of capture years compared to CHS (9 vs. 4). Both populations consisted mainly of adult dolphins (73.5% IRL, 67.5% CHS). The sex distributions for the IRL and CHS populations were virtually identical; 62.6 and 61.4 percent male, respectively. Thus, with respect to their demographic characteristics the populations were comparable.

For the purposes of this review, the salient features of the disease prevalence data are summarized here. Details are provided elsewhere (3). During the first 5 years of the study (2003–2007) the prevalence of disease was 32.5% in the IRL (*n* = 140) and 21.1% at CHS (*n* = 90). Disease prevalence increased at both sites during this period. The second observation period spanned the years 2010 to 2015 with only a single capture year at CHS (2013). The prevalence of disease remained relatively stable at both sites (29.2% IRL, 36.8% CHS) indicating high endemic morbidity rates.

Overall, the major contributors to morbidity in both populations were infectious diseases. Specifically, the prevalence of orogenital papillomatosis was high in both populations and accounted for approximately two thirds of disease diagnoses among first captures in both populations. Increases in morbidity during the first 3 years of the study in the CHS population were mainly attributable to OP. The epidemic curve for OP at CHS resembled epidemic propagation in a naïve population with prevalence increasing three-fold (9% in 2003 to 33% in 2005) (3). Limited prior exposure to the novel papillomaviruses and herpesviruses that cause OP could have been responsible for the rapid expansion of this sexually transmitted neoplastic disorder.

The second major component of infectious disease morbidity was PC caused by a recently characterized strain of *P. brasiliensis* (62). This fungal disease is endemic in the IRL, with a prevalence of 11 percent over the 13 years of observation. PC has not been observed in the CHS population. Within the IRL, the disease follows a spatial gradient with the highest rates in the southern reaches of the estuary (59, 72, 73) for reasons which remain to be elucidated.

In summary, both estuarine dolphin populations have a high prevalence of morbidity, the preponderance of which is due to infectious diseases. In all likelihood, environmental stressors and concurrent perturbations in immune function play a role in this constellation of endemic disease (3, 4).

### Immune Parameters

The immune parameters evaluated included leukocyte hematology, innate, and adaptive immune measurements, antibody titers to common marine bacteria and serum protein electrophoresis. Tables 1–4 summarize the comparisons between dolphins seropositive for CeMV and *Chlamydiaceae* and dolphins with biopsy-confirmed PC and OP and healthy dolphins, respectively. The tables were adapted from previously published data (17–19, 25). Wide ranges of host immunologic responses were found with immunopathologic or serologic evidence of infection with these specific viral, bacterial, and fungal pathogens.

**Table 1:** Immune parameters in Atlantic bottlenose dolphins (*Tursiops truncatus*) with positive cetacean morbillivirus (CeMV) antibody titers and seronegative CeMV healthy dolphins.

**Immune parameter**	**Seronegative healthy dolphins (*****n*** **=** **49)**		**Seropositive CeMV dolphins (*****n*** **=** **14)**		
	**Mean**	**SD**	**Mean**	**SD**	***p*-value**
**LEUKOCYTE HEMATOLOGY**					
Total White Blood Cells (10^3^cells/ul)	9.65	1.79	10.89	3.31	0.21
Lymphocytes (10^3^cells/ul)	1.84	0.74	1.78	1.08	0.80
Segmented Neutrophils (10^3^cells/ul)	4.12	1.09	4.70	1.20	0.09
Eosinophils (10^3^cells/ul)	3.33	1.26	3.99	1.77	0.13
**INNATE IMMUNITY**					
Granulocytic Phagocytosis (%)	19.77	10.60	23.41	10.42	0.36
Monocytic Phagocytosis (%)	18.38	10.28	26.18	15.43	0.08
Natural Killer Cell Activity (100:1)	13.70	10.92	14.68	15.33	0.83
Lysozyme Concentration (ug/ul)	6.94	2.50	8.84	2.86	**0.03**
**ADAPTIVE IMMUNITY**					
CD2 T Cells (Absolute Nos.)	737.18	469.73	612.23	420.82	0.45
CD4 Helper T Cells (Absolute Nos.)	346.06	218.90	217.10	123.10	0.08
CD 19 B Cells-Immature (Absolute Nos.)	373.40	250.14	231.69	231.45	0.11
CD 21 B Cells-Mature (Absolute Nos.)	462.61	276.16	317.37	285.30	0.25
CD2/CD4 Ratio	2.07	0.68	2.62	1.34	0.24
CD2/CD21 Ratio	1.85	1.56	3.21	2.93	0.31
MHCII+ (Absolute Nos.)	1272.02	649.52	921.33	504.94	0.11
T Cell Proliferation (Con A 2.5)	602.61	488.21	329.17	239.64	**0.01**
B Cell Proliferation (LPS 120)	84.10	147.57	32.22	57.27	0.22
**ANTIBODY TITERS (U/ul) TO COMMON MARINE BACTERIA**					
*Escherichia coli*	146.02	126.26	108.99	137.04	0.43
*Erysipelas rhusiopathiae*	133.52	116.38	99.02	125.30	0.42
*Mycobacterium marinarum*	158.77	142.71	119.91	160.06	0.46
*Vibrio cholera*	144.77	131.63	114.68	148.75	0.54
*Vibrio parahaemolyticus*	158.40	144.23	131.24	164.26	0.61
**SERUM PROTEIN ELECTROPHORESIS**					
Total Protein (g/dl)	7.43	0.41	7.52	0.55	0.41
Albumin (g/dl)	4.58	0.26	4.36	0.27	**0.01**
Total Globulin (g/dl)	3.74	0.50	3.87	0.60	0.44
A/G Ratio	1.64	0.29	1.42	0.28	**0.02**
Total Alpha Globulin (g/dl)	1.27	0.19	1.17	0.26	0.11
Alpha-1 globulin (g/dl)	0.33	0.15	0.27	0.14	0.24
Alpha-2 globulin (g/dl)	0.94	0.16	0.89	0.24	0.48
Total Beta Globulin (g/dl)	0.44	0.07	0.50	0.13	0.06
Gamma Globulin (g/dl)	2.85	0.41	3.16	0.56	**0.03**

*Adapted from Bossart et al. (18). Bold values represent statistically significant values at p < 0.05*.

**Table 2:** Immune parameters in Atlantic bottlenose dolphins (*Tursiops truncatus*) with positive *Chlamydiaceae* antibody titers and seronegative *Chlamydiaceae* healthy dolphins.

**Immune parameter**	**Seronegative healthy dolphins (*****n*** **=** **83)**		***Chlamydiaceae*** **seropositive dolphins (*****n*** **=** **43)**		
	**Mean**	**SD**	**Mean**	**SD**	***p*-value**
**LEUKOCYTE HEMATOLOGY**					
Total White Blood Cells (10^3^cells/ul)	10.55	0.33	10.90	0.44	0.48
Lymphocytes (10^3^cells/ul)	2.24	0.25	3.27	0.41	**0.05**
Segmented Neutrophils (10^3^cells/ul)	4.29	0.86	8.36	1.45	**0.03**
Eosinophils (10^3^cells/ul)	3.81	0.60	6.06	1.10	0.07
**INNATE IMMUNITY**					
Granulocytic Phagocytosis (%)	23.74	1.83	17.91	2.21	**0.05**
Monocytic Phagocytosis (%)	18.65	1.71	21.67	2.08	0.28
Natural Killer Cell Activity (100:1)	6.80	1.26	11.53	1.56	**0.02**
Lysozyme Concentration (ug/ul)	6.22	0.41	8.84	0.54	** < 0.01**
**ADAPTIVE IMMUNITY**					
CD2 T Cells (Absolute Nos.)	867.09	77.77	856.90	11.24	0.96
CD4 Helper T Cells (Absolute Nos.)	387.79	34.79	436.40	46.32	0.38
CD 19 B Cells-Immature (Absolute Nos.)	519.71	54.01	427.91	83.89	0.35
CD 21 B Cells-Mature (Absolute Nos.)	708.98	82.62	1129.81	162.07	**0.03**
CD2/CD4 Ratio	2.50	1.17	2.40	1.27	0.30
CD2/CD21 Ratio	1.48	0.20	1.85	1.09	0.39
MHCII+ (Absolute Nos.)	1565.91	126.71	1909.70	183.35	0.13
T Cell Proliferation (Con A 2.5)	551.64	47.32	356.82	67.22	**0.02**
B Cell Proliferation (LPS 120)	111.97	17.31	25.83	23.66	** < 0.01**
**ANTIBODY TITERS (U/ul) TO COMMON MARINE BACTERIA**					
*Escherichia coli*	151.28	21.28	182.52	84.29	0.77
*Erysipelas rhusiopathiae*	143.80	20.20	309.24	76.36	**0.05**
*Mycobacterium marinarum*	142.04	19.83	234.54	77.29	0.28
*Vibrio cholerae*	155.50	122.04	223.60	84.88	0.44
*Vibrio parahaemolyticus*	166.67	23.99	204.96	92.61	0.75
**SERUM PROTEIN ELECTROPHORESIS**					
Total Protein (g/dl)	7.25	0.07	7.20	0.09	0.73
Albumin (g/dl)	3.64	0.044	3.70	0.05	0.38
Total Globulin (g/dl)	2.78	0.07	2.90	0.09	0.31
A/G Ratio	1.73	0.59	1.51	0.35	0.21
Total Alpha Globulin (g/dl)	1.31	0.03	1.13	0.03	** < 0.01**
Alpha-1 globulin (g/dl)	0.39	0.02	0.39	0.03	0.94
Alpha-2 globulin (g/dl)	0.92	0.03	0.74	0.04	** < 0.01**
Total Beta Globulin (g/dl)	0.46	0.01	0.44	0.01	0.38
Gamma Globulin (g/dl)	1.78	0.06	1.95	0.09	0.12

*Adapted from Bossart et al. (19). Bold values represent statistically significant values at p < 0.05*.

**Table 3:** Immune parameters in Atlantic bottlenose dolphins (*Tursiops truncatus*) with paracoccidioidomycosis ceti (PC) and healthy dolphins without PC.

**Immune Parameter**	**Healthy dolphins (*****n*** **=** **40)**		**Dolphins with PC (*****n*** **=** **8)**		
	**Mean**	**SD**	**Mean**	**SD**	***p*-value**
**LEUKOCYTE HEMATOLOGY**					
Total White Blood Cells (10^3^cells/ul)	9.65	1.80	10.64	2.78	0.68
Lymphocytes (10^3^cells/ul)	2.00	1.00	1.18	0.84	**0.02**
Segmented Neutrophils (10^3^cells/ul)	4.10	0.90	5.98	2.24	**0.02**
Eosinophils (10^3^cells/ul)	3.26	1.36	2.88	0.82	0.62
**INNATE IMMUNITY**					
Granulocytic Phagocytosis (%)	19.9	10.5	18.3	12.0	0.81
Monocytic Phagocytosis (%)	19.1	10.1	19.7	5.7	0.95
Natural Killer Cell Activity (100:1)	10.9	9.7	17.0	12.3	0.23
Lysozyme Concentration (ug/ul)	7.3	2.9	11/.2	4.6	**0.02**
**ADAPTIVE IMMUNITY**					
CD2 T Cells (Absolute Nos.)	911.4	620.9	660.5	683.3	0.07
CD4 Helper T Cells (Absolute Nos.)	389.1	237.5	188.3	144.4	**0.02**
CD 19 B Cells-Immature (Absolute Nos.)	366.9	278.6	160.3	180.8	**0.03**
CD 21 B Cells-Mature (Absolute Nos.)	534.4	334.3	199.6	228.3	**0.01**
CD2/CD4 Ratio	2.37	0.63	3.36	1.18	**0.05**
CD2/CD21 Ratio	2.34	1.63	5.63	3.29	**0.01**
MHCII+ (Absolute Nos.)	1445.0	822.0	870.0	839.5	**0.03**
T Cell Proliferation (Con A 2.5)	615.4	454.4	264.6	276.4	**0.03**
B Cell Proliferation (LPS 120)	93.5	156.7	34.4	72.2	**0.01**
**ANTIBODY TITERS (U/ul) TO COMMON MARINE BACTERIA**					
*Escherichia coli*	113.9	126.4	3.7	1.7	0.09
*Erysipelas rhusiopathiae*	104.2	116.4	2.8	1.2	**0.04**
*Mycobacterium marinarum*	126.3	144.3	7.8	5.6	0.09
*Vibrio cholerae*	113.7	127.2	4.4	1.8	0.10
*Vibrio parahaemolyticus*	126.5	140.4	13.9	18.9	0.26
**SERUM PROTEIN ELECTROPHORESIS**					
Total Protein (g/dl)	7.27	0.41	7.96	0.87	**0.02**
Albumin (g/dl)	4.51	0.24	4.22	0.37	**0.04**
Total Globulin (g/dl)	3.58	0.48	4.27	0.77	**0.01**
A/G Ratio	1.06	0.21	0.88	0.16	**0.04**
Total Alpha Globulin (g/dl)	1.20	0.22	1.29	0.18	0.30
Alpha-1 globulin (g/dl)	0.32	0.17	0.52	0.25	**0.03**
Alpha-2 globulin (g/dl)	0.88	0.17	0.77	0.30	0.38
Total Beta Globulin (g/dl)	0.44	0.10	0.50	0.09	**0.05**
Gamma Globulin (g/dl)	1.94	0.44	2.49	0.70	**0.03**

*Adapted from Reif et al. (25). Bold values represent statistically significant values at p < 0.05*.

**Table 4:** Immune parameters in Atlantic bottlenose dolphins (*Tursiops truncatus*) with orogenital papillomas (OP) and healthy dolphins without OP.

**Immune Parameter**	**Healthy dolphins (*****n*** **=** **86)**		**Dolphins with OP (*****n*** **=** **22)**		
	**Mean**	**SD**	**Mean**	**SD**	***p*-value**
**LEUKOCYTE HEMATOLOGY**					
Total White Blood Cells (10^3^cells/ul)	10.13	1.86	10.24	2.22	0.74
Lymphocytes (10^3^cells/ul)	2.32	1.20	2.12	0.70	0.75
Segmented Neutrophils (10^3^cells/ul)	3.85	1.00	4.54	1.65	0.11
Eosinophils (10^3^cells/ul)	3.63	1.51	3.27	1.26	0.34
**INNATE IMMUNITY**					
Granulocytic Phagocytosis (%)	20.5	11.7	30.3	12.4	** < 0.01**
Monocytic Phagocytosis (%)	19.2	13.1	28.8	13.6	** < 0.01**
Natural Killer Cell Activity (100:1)	14.21	12.96	10.17	8.83	0.36
Lysozyme Concentration (ug/ul)	6.45	2.55	6.14	1.85	0.97
**ADAPTIVE IMMUNITY**					
CD2 T Cells (Absolute Nos.)	861.3	531.4	774.0	432.0	0.66
CD4 Helper T Cells (Absolute Nos.)	397.0	220.1	355.5	178.0	0.54
CD 19 B Cells-Immature (Absolute Nos.)	569.1	622.1	447.4	225.0	0.88
CD 21 B Cells-Mature (Absolute Nos.)	867.4	917.2	584.8	352.3	0.40
CD2/CD4 Ratio	2.24	0.81	2.43	1.36	0.96
CD2/CD21 Ratio	1.68	1.34	1.76	1.42	0.54
MHCII+ (Absolute Nos.)	1731.7	1141.3	1254.2	683.5	0.07
T Cell Proliferation (Con A 2.5)	599.9	419.8	581.8	344.3	0.94
B Cell Proliferation (LPS 120)	99.3	155.3	173.5	164.2	** < 0.01**
**ANTIBODY TITERS (U/ul) TO COMMON MARINE BACTERIA**					
*Escherichia coli*	129.9	130.2	250.7	25.9	** < 0.01**
*Erysipelas rhusiopathiae*	117.8	117.8	218.1	35.2	**0.03**
*Mycobacterium marinarum*	147.5	156.0	312.6	82.6	** < 0.001**
*Vibrio cholera*	120.8	121.9	231.3	58.1	** < 0.01**
*Vibrio parahaemolyticus*	142.7	140.8	281.4	37.1	** < 0.01**
**SERUM PROTEIN ELECTROPHORESIS**					
Total Protein (g/dl)	7.15	0.45	7.31	0.47	0.35
Albumin (g/dl)	4.52	0.25	4.42	0.25	0.15
Total Globulin (g/dl)	2.62	0.54	2.85	0.56	**0.03**
A/G Ratio	1.84	0.61	1.62	0.37	0.13
Total Alpha Globulin (g/dl)	1.25	0.23	1.36	0.15	**0.02**
Alpha-1 globulin (g/dl)	0.40	0.21	0.36	0.15	0.70
Alpha-2 globulin (g/dl)	0.85	0.26	1.01	0.17	**0.02**
Total Beta Globulin (g/dl)	0.46	0.10	0.47	0.06	0.26
Gamma Globulin (g/dl)	1.69	0.54	1.85	0.49	0.31

*Adapted from Bossart et al. (17). Bold values represent statistically significant values at p < 0.05*.

### White Blood Cell Hematology

Dolphins seropositive to *Chlamydiaceae* (as defined above) had higher absolute numbers of neutrophils and lymphocytes compared to healthy dolphins. Conversely, dolphins with PC were shown to have higher absolute numbers of neutrophils and lower absolute numbers lymphocytes compared to healthy dolphins. For CeMV infection and OP, total WBC counts and WBC differential counts were not significantly different when compared with healthy dolphins.

### Innate Immunity

Lysozyme activity was significantly increased in dolphins seropositive to CeMV and *Chlamydiaceae* and in dolphins with PC compared to healthy dolphins. Granulocytic and monocytic phagocytosis were significantly increased with OP. Monocytic phagocytosis was also increased in CeMV seropositive dolphins but not significantly. In contrast, granulocytic phagocytosis was significantly decreased and NK activity increased in *Chlamydiaceae seropositive* dolphins.

### Adaptive Immunity

Substantial alterations in parameters of adaptive immunity were found in dolphins affected with all four of the disorders under study. Dolphins seropositive to CeMV had a significant reduction in T cell lymphocyte proliferation compared to healthy dolphins. Both T and B cell responses were reduced in dolphins seropositive to *Chlamydiaceae* and those with clinical and histologic evidence of PC. Conversely, B lymphocyte proliferation was significantly higher in dolphins with OP.

Alterations were also found in lymphocyte surface markers in affected dolphins. Dolphins seropositive for *Chlamydiaceae* had higher absolute numbers of CD 21 mature B lymphocytes. In contrast, dolphins with PC had significant reductions in multiple markers including absolute numbers of CD4+ helper T cells, CD19+ immature B cells, and CD21+ mature B cells. The absolute number of lymphocytes expressing the MHC class II molecule was also reduced in dolphins with PC. Increases in the ratios of CD2+ to CD4+ cells and CD2+ to CD21+ cells were also noted in dolphins with this fungal disease. CeMV seropositive dolphins had a marginally significant reduction in the absolute number of CD4+ helper T cells (*p* = 0.08).

### Antibody Titers to Common Marine Bacteria

The antibody titers to the common marine microorganisms *E. coli, M. marinum, V. cholerae, V. parahemolyticus* and *E. rhusiopathiae* were dramatically reduced in dolphins with PC (Table 3) although only the latter was statistically significant. In dolphins with OP, in contrast, antibody titers to all five organisms were significantly increased. Antibody concentrations to these organisms were also generally higher in dolphins seropositive for *Chlamydiaceae*, but only the mean titer against *E. rhusiopathiae* reached statistical significance.

### Serum Protein Electrophoresis

Protein abnormalities were present suggesting immunologic disturbances in CeMV seropositive dolphins and dolphins with PC and OP. CeMV seropositive dolphins and dolphins with PC had a significant hypergammaglobulinemia with a resultant decreased A:G ratio. PC dolphins also had concurrent significant elevations in alpha-1 globulins and total beta globulins resulting in a significantly elevated total protein. Dolphins with OP had a significantly elevated alpha-2 globulins resulting in a significantly elevated total alpha globulins and total globulins. *Chlamydiaceae sero*positive dolphins had significantly lower alpha-2 globulins titers and resultant total alpha globulins, which are probably not immunologically relevant. Albumin levels were significantly lower in CeMV seropositive and PC dolphins. However, the albumin levels are within the normal range for this species and thus are likely not clinically relevant.

## Discussion

In general, a wide range of immunologic host responses was associated with each specific pathogen, which reflects the dynamic and complex interactions that occur between the innate and adaptive arms of the immune system. The host responses of the bottlenose dolphin to an array of bacterial, fungal, and viral pathogens provide a novel glimpse of the immune system function for this comparative marine mammal model.

CeMV seropositive dolphins had a reduced adaptive immune response as shown by a decreased T lymphocyte proliferation response to concanavalin A and a marginally significant reduction in absolute numbers of CD4+ lymphocytes compared to healthy dolphins. An up-regulation of the innate immune response occurred concurrently, with significant increases in lysozyme concentration and a marginally significant increase in monocytic phagocytosis.

The observed pattern of impaired cell-mediated immunity in CeMV infection resembles the response to acute morbillivirus infection described in other species; e.g., measles in humans, canine distemper virus in the dog (18). CeMV seropositive dolphins had a significant hypergammaglobulinemia with a resultant decreased A:G (albumin:globulin) ratio indicating an upregulated adaptive humoral immune response. Interestingly, high levels of the gamma globulin IgG are found in dogs with canine distemper that recover from disease (74). However, a prolonged immune system response resulting in persistent chronic inflammation and elevations of immunoglobulin could also be deleterious to health and contribute to a variety of chronic inflammatory diseases (75, 76). Subclinical CeMV infection in IRL dolphins appears to be accompanied by some degree of cell-mediated immunosuppression. This reduced component of immune surveillance could render this population more susceptible to opportunistic infections and may have contributed to the frequent UMEs reported in bottlenose dolphins in this area (18, 50). Opportunistic infections may also have contributed to the elevated levels of gamma globulins as originally hypothesized (18).

Subclinical infection with *Chlamydiaceae* also appeared to induce multiple complex immunologic abnormalities (19). A significant absolute neutrophilia and absolute lymphocytosis were present in seropositive dolphins suggesting a granulocytic and monocytic peripheral blood response to this bacterial infection. The results further suggest a mixed response of the adaptive and innate arms of the immune system. *Chlamydiaceae* seropositive dolphins had lower T and B lymphocyte proliferative responses to mitogens compared to healthy controls. However, the absolute numbers of CD21 mature B cells were increased. In terms of innate immune responses, lysozyme concentration and NK activity were higher in seropositive dolphins suggesting up-regulation, while granulocytic phagocytosis was lower. These results are not entirely consistent but overall suggest a complex immunologic response and possible functional impairment of both humoral and cell-mediated immunity.

Comparatively, the immune responses in humans and laboratory animals with *Chlamydiaceae* infection are dynamic and similarly complex. The unique extracellular infectious and intracellular vegetative phases of the pathogen can produce various immune-mediated responses that may lead to disease resolution or progression (51, 53). Innate responses to *Chlamydiaceae* infection include initial local recruitment of leukocytes to the site of infection and the production of pro-inflammatory cytokines and chemokines including lysozyme (77). The adaptive immune response in *Chlamydiaceae* infections can limit the spread of infection and protect against recurrent infections. For example, upon *C. trachomatis* infection, CD4+ cells become activated, begin to proliferate with a characteristic Th1 response, secreting large amounts of interferon γ required to aid in clearing bacterial infection (78). B cells are able to modulate immunity during *Chlamydiaceae* infection by several mechanisms including antibody-mediated neutralization, antibody-dependent cellular cytotoxicity, and the formation of antibody–antigen complexes that bind to receptors on antigen presenting cells (79).

Dolphins with serologic evidence of *Chlamydiaceae* infection had a significant increase in their antibody titers against E*. rhusiopathiae*. A potential explanation for this association lies in the fact that marine birds have been shown to be infected with both *E. rhusiopathiae* and *Chlamydophila psittaci* (80). Both estuarine locales have abundant avian wildlife. Thus, the high prevalence of antibody to both agents may be due to extensive shedding in feces and nasal discharges by local bird reservoirs with resultant infection of bottlenose dolphins in these shallow marine environments. Although clinical chlamydiosis has not been reported in the IRL or CHS dolphin population, exposure to *Chlamydiaceae* appears to be prevalent, as supported by serologic data in other marine mammal populations (19) and our previous results (26). Thus, subclinical infections with both *Chlamydiaceae* and CeMV infections appear to affect immune status which may in turn contribute to an increased risk for opportunistic infection and a decline in population health.

Perhaps the most profound and complex significant immunologic responses were found in dolphins with clinical and histopathologic evidence of PC (historically termed lobomycosis/lacaziasis) now known to be caused by a novel strain of *P. brasiliensis*. A significant absolute neutrophilia with significant elevations of lysozyme activity were present in dolphins with PC, which suggest a systemic response to a bacterial or fungal infection. Secondary bacterial infection is often a complication with cutaneous ulcerative disease in dolphins including those with fungal infections (9, 81). Dolphins with this fungal disorder had a marked depression of the adaptive immune response. T and B lymphocyte proliferative responses were significantly reduced as were antibody titers to *E. rhusiopathiae*. These changes were accompanied by statistically significant decreases in the absolute numbers of peripheral blood lymphocytes, and in lymphocyte expression of MHC class II molecules, CD4+ helper T cells, and CD19+ and CD21+ B cells. Increases in the CD2+/CD4+ and CD2+/CD21+ ratios were also present compared to healthy controls. Dolphins with PC also had a significant hypergammaglobulinemia with a resultant decreased A:G ratio suggesting an immunoglobulin response to systemic infection. At first glance, the presence of hypergammaglobulinemia with concurrent decreases in immature and mature B lymphocytes and reduced B lymphocyte proliferation appears counterintuitive. Hypogammaglobulinema might be expected as a downstream effect of the other adaptive humoral immune parameter decreases. The significance of this observation is unknown. The elevations in alpha-1 globulins and total beta globulins in PC dolphins resulted in a significantly elevated total protein, which suggests an acute phase protein response to the fungal infection.

Historically, it was believed that humans and dolphins were the only species that developed the disease known as lobomycosis. The clinical and histologic features of the disease in humans and dolphins are remarkably similar (59). However, newer knowledge indicates that the infection in the dolphin is caused by an organism closely related to *P. brasiliensis*, the causal agent of human paracoccidioidomycosis in South America (62). Human lobomycosis is still considered to be caused by *Lacazia* loboi. Therefore, reviewing the pathogenesis of paracoccidioidomycosis may provide helpful clues toward understanding the immune dysfunction in dolphin PC. Humans infected with the classical strain of *P. brasiliensis* experience immunologic alterations that switch the host response to the pathogen from a Th1 to a Th2 profile (82). Histologically, the dermal lesions in dolphins with PC consist of granulomatous inflammation with infiltration of macrophages, epithelioid cells, and multinucleated giant cell formation. Yeast-like cells occurring singly or in chains are found within the granulomas as described above (9, 20, 59, 64). The abundance of organisms within the lesions suggests that the local immune response is ineffective in destroying the organisms and preventing lesion progression in PC. Similarly, in human lobomycosis, there is dense, granulomatous inflammation with infiltration of macrophages, CD4+ T cells, multinucleated giant cells, and viable yeast cells (83). Quantitative analysis of cytokine expression in peripheral blood mononuclear cell cultures obtained from patients with lobomycosis was consistent with a Th2 response (84). Subsequent quantification of cytokine profiles in skin lesions showed that TGF- ß1 and IL-10 were the predominant cytokines expressed (83). The authors therefore suggested that the failure to eliminate the pathogen was due to the presence of these immunosuppressive cytokines within lesions which predominate in the Th2 response. Thus, it seems plausible that a similar switch to a Th2 response occurs in PC, as shown in human paracoccidioidomycosis and lobomycosis and may be responsible for maintaining the organism in dolphin skin lesions by inhibiting phagocytic cell functions. Whichever mechanism is correct, observational evidence from our long-term follow-up of chronically infected dolphins indicates that those animals with widely disseminated lesions have a poor prognosis and are likely to succumb after several months to years. The underlying severe decrease in adaptive immunity is likely to be a major factor in their demise (25). In contrast, although a decrease in markers of cell-mediated immunity has been described in human lobomycosis (85), the lesions remain localized and do not appear to impact survival. The difference in prognosis between humans and dolphins may be attributed to their unique etiologies, and immunologic responses.

Dolphins with OP showed an upregulation of multiple parameters of innate immunity. Increases in granulocytic and monocytic phagocytic activity were demonstrated in affected dolphins compared to controls. There was also evidence of upregulated adaptive humoral immunity as shown by higher B lymphocyte proliferation in response to lipopolysaccharide and higher concentrations of antibodies to *E. coli, E. rhusiopathiae, M. marinum, V. cholerae*, and *V. parahemolyticus*. Hyperglobulinemia and hyperalphaglobulinemia were also present in affected dolphins. The combined immune upregulation likely represented acute phase inflammatory and adaptive humoral immune responses to either the papilloma or herpes viruses present in the lesions or to constituents of the tumors themselves (17, 86, 87). It is also possible that dolphins with OP have increased exposure to other pathogens based on the combined upregulated immune responses, concurrent higher concentrations of antibodies to marine bacteria and the sexually-transmitted nature of the disease.

In contrast, in humans with PV-associated genital lesions, the viral infection down regulates the innate immune signaling pathways and this evasion of innate immune system recognition appears to be an important factor in disease pathogenesis (88). Regression of human PV-associated genital lesions is accompanied by an effective cell-mediated CD4^+^ T cell-dominated Th1 response and failure to develop an effective cell-mediated response results in persistent infection and an increased probability of progression to invasive carcinoma (89). The basis for the differences in immunologic response between human and dolphin PV-associated infections remains unclear.

Several limitations of the studies reviewed here should be mentioned. First, two of the conditions were based on clinical appearance and were histologically confirmed (PC, OP). CeMV and *Chlamydiaceae* infection were assessed serologically. Lacking evidence from isolation and culture or molecular identification of the pathogen, the status of infection, the duration of infection, and other components of the infectious disease process could not be assessed adequately with respect to the immunologic status of the animal.

Second, there is potential for misclassification since several dolphins with CeMV also had PC (3) or OP (4). Generally, the effects of co-morbidity with PC could have been to augment those of CeMV while those of OP could have diminished the overall responses. With a small sample size, the effect of this misclassification cannot be quantitated.

Finally, the IRL and CHS study sites represent unique ecosystems that are impacted differentially by anthropogenic contaminants and other environmental stressors. Neither site represents a pristine, non-impacted environment. The IRL is heavily contaminated with mercury as the result of atmospheric deposition through rainfall (4). The concentrations of total mercury in the blood and skin of IRL dolphins are among the highest reported worldwide and approximately five times higher than in CHS dolphins (4). Conversely, the concentrations of persistent organic pollutants such as polychlorinated biphenyl compounds, polybrominated diphenyl ethers, perfluorinated compounds, and pesticides including DDT are higher in the blubber of CHS dolphins than those in the IRL. The role of these contaminants on immune function, singularly and in combination, was not assessed. By selecting healthy controls from each study site in the same proportions as the cases, the potential effects of these exposures are controlled for at the group level.

## Conclusions

This represents the first comparative review documenting the wide range of innate and adaptive immune responses to specific viral, bacterial, and fungal infectious diseases in free-ranging dolphins as described in our previous publications. The data demonstrate the intricate, complex and dynamic interactions that occur between infectious disease and immunologic responses in this species. Additionally, the evaluation of the type, pattern, and degree of immunologic responses to these pathogens provides novel insight on immunopathogenesis. In some cases, infection may be associated with immunopathologic perturbations that could affect overall individual and population health. Specifically, either chronic immune system activation or down-regulation could lead to eventual immunologic dysfunction and the inability to eliminate chronic inflammation. In other mammals, including humans, persistent chronic inflammation increases the risk for cancer, autoimmune disease, and cardiovascular disease and increases vulnerability to infectious disease. The studies summarized here add to the body of evidence that estuarine bottlenose dolphins are an excellent sentinel for the ecosystem and may serve as an early warning system for emerging diseases that can affect human health.

## Author Contributions

GB and JR were responsible for preparing and organizing written manuscript drafts and wrote the manuscript. TR, MP-A, AS, PF, and CR were responsible for adding methodology details and specific data interpretation in their particular area of expertise and participated in interpreting the results. All authors reviewed the final version of the manuscript and agreed to its submission.

### Conflict of Interest Statement

The authors declare that the research was conducted in the absence of any commercial or financial relationships that could be construed as a potential conflict of interest.
